# Health related quality of life among adolescents with premenstrual disorders: a cross sectional study

**DOI:** 10.1186/1477-7525-10-1

**Published:** 2012-01-01

**Authors:** Mahin Delara, Fazlollah Ghofranipour, Parviz Azadfallah, Sedigheh Sadat Tavafian, Anoushirvan Kazemnejad, Ali Montazeri

**Affiliations:** 1 Depratment of Health Education, Medical School, Tarbiat Modares University, Tehran, Iran; 2 Department of Biostatistics, Medical School, Tarbiat Modares University, Tehran, Iran; 3 Mental Health Research Group, Health Metrics Research Centre, Iranian Institute for Health Sciences Research, ACECR, Tehran, Iran

## Abstract

**Background:**

Premenstrual disorders usually refer to premenstrual syndrome (PMS) and premenstrual dysphoric disorder (PMDD). This study was designed to evaluate health-related quality of life (HRQOL) in a sample of Iranian adolescents with premenstrual disorders.

**Methods:**

This was a cross sectional study. A sample of adolescent schoolgirls aged between 14 and 19 years were included in the study. Premenstrual disorders were indicated according to the International Classification of Diseases (ICD-10) and the Diagnostic and Statistical Manual of Mental Disorders (DSM-IV). Health-related quality of life was measured using the Short Form Health Survey (SF-36). The data were analyzed in a descriptive fashion and were compared among subgroups of the study sample.

**Results:**

In all 602 female students were studied. All students reported at least one premenstrual symptom. Of these, 224 (37.2%) met the diagnostic criteria for premenstrual dysphoric disorder (PMDD). Comparing the SF-36 scores between female students with and without PMDD, it was found that there were significant differences between these two groups in all measures (P < 0.001) except for physical functioning (P = 0.274). These differences were more evident on role emotional, role physical, social functioning and bodily pain.

**Conclusion:**

The study findings affirm the fact that adolescents with premenstrual disorders suffer from poor health-related quality of life. In order to improve quality of life in female adolescents appropriate support should be provided for this population especially for those who suffer from more severe premenstrual disorders.

## Background

Premenstrual disorders namely premenstrual syndrome (PMS) and premenstrual dysphoric disorder (PMDD), are a group of physical, cognitive, affective, and behavioral symptoms that occur cyclically during the luteal phase of the menstrual cycle and resolve at or within a few days of the onset of menstruation [1]. The common symptoms of PMS and PMDD include swelling, breast tenderness, aches, headache, bloating, sleep disturbances, appetite change, poor concentration, decreased interest, social withdrawal, irritability, mood swings, anxiety/tension, depression, and feeling out of control [2]. Of these, six symptoms identified as core symptoms suggesting that clinical diagnosis of PMS can be developed around a core symptom group. The identified core symptoms are: anxiety/tension, mood swings, aches, appetite/food cravings, cramps, and decreased interest in activities [3]. However, although it has been estimated that a high proportion of women in reproductive age (up to 90%) experience some degree of premenstrual symptoms, the diagnosis of PMS or PMDD is assigned to those women whose lives are significantly affected by moderate to severe symptoms [4]. As noted by Rapkin and Mikacich premenstrual disorders likely start in the teen years and at least 20% of adolescents may experience moderate-to-severe premenstrual symptoms. They indicated that the literature suggests that a similar proportion of teens would also meet criteria for PMS/PMDD [4,5].

Premenstrual symptoms might cause several difficulties for women including impairment in physical functioning, psychological health and severe dysfunction in social or occupational realms [6]. In young adolescents symptoms might particularly affect school functions, and social interactions in a negative way [7]. Previous studies have also shown that women with premenstrual disorders have a poor health-related quality of life [8-10]. In a comprehensive review of the literature Parkin and Winer distinguished four types of studies that evaluated the PMS/PMDD effect on health-related quality of life and for instance reported that 'the affective, behavioral and physical symptoms of PMDD have been shown to adversely affect health-related quality of life to a disabling degree, especially regarding interpersonal relationships with family members and partner' or 'women with PMDD suffer impairment that is as severe as women with chronic clinical depression and that their luteal phase adjustment to social and leisure activities is even worse than women with other types of depression' [9].

It is believed that improvements in HRQOL reduces the complications associated with this disorder, or at least makes it more tolerable. To attain this goal, we must first assess quality of life in adolescents in order to tailor the concept of HRQOL to this population and integrate these into a comprehensive plan for care. The purpose of this study was to assess health-related quality of life (HRQOL) in a sample of Iranian adolescents with premenstrual disorders (PMS and PMDD). It was hoped that this preliminary study could contribute to the existing knowledge on the topic and provide necessary information for possible future interventions.

## Methods

### The study sample and design

A cross sectional study was conducted in Sabzevar boarding high schools. Sabzevar is a city in Khorasan province, located in the east of Iran. There are only three boarding high schools in the city. All students in these high schools were invited to participate in the research. Students with irregular menstrual cycles, current major medical and psychological problems, those receiving hormonal therapy and experiencing a catastrophe shortly before or during the study were excluded from the study.

### Quality of life measure

Quality of life was measured using the Short Form Health Survey (F-36). The SF- 36 is a generic instrument that consists of eight subscales: physical functioning, role physical, bodily pain, general health, vitality, social functioning, role emotional, and mental health. Scores in each subscale range from zero to 100, with zero representing the worst HRQOL and 100 representing the best possible score. Previous evaluations of the original as well as the Persian version of the SF-36 indicated good reliability and construct validity [11].

### Additional measures

1. The Symptom Check List (SCL-90-R) [12] to exclude students with probable severe psychological disorders due to other reasons. As such students with score greater than 63 (the cut-off point on the SCL-90-R) considered to suffer from severe depression and anxiety and were excluded from the study.

2. A designed questionnaire based on the ICD-10 for PMS diagnosis. According to the ICD-10 a female suffering from at least one distressing premenstrual symptom could be regarded as having PMS [13].

3. A designed questionnaire based on Diagnostic and Statistical Manual, 4th edition (DSM-IV) for PMDD diagnosis [14].

4. A questionnaire for collecting data on demographic and menstrual characteristics of the study sample. This questionnaire consisted of 2 parts. Part 1 included questions about socio-demographic information such as age, marital status, parents' employment, and economic status. Part 2 included questions about menstrual characteristics such as the severity of menstrual bleeding, the length of menstrual bleeding, menstrual cycle duration, the presence of dysmenorrhea and age of menarche. An additional question was also designed in this part to evaluate the awareness of the students about premenstrual disorders.

### Statistical analysis

The data were analyzed in a descriptive fashion. Also univariate analysis of variance was performed to compare age adjusted quality of life data between adolescents with and without PMDD.

### Ethics

The Ethics Committee of Tarbiat Modares University approved the study. All participants gave their written consent.

## Results

In all there were 636 female students. Of these, 18 students were absent. All the remaining students (n = 618) agreed to participate in the study. However, 602 students (94.6%) met the inclusion criteria. The mean age of participants was 15.78 (SD = 1.06) years (ranging from 14 to 19 years). The mean age of menarche was 12.99 (SD = 1.13) years. The mean duration of menstrual bleeding was 7.2 (SD = ± 3.31) days and the mean length of menstrual cycle was 27.1 (SD = ± 6.5) days. Students scores on the Symptom Check List (SCL-90-R) was 1.45 (SD = 0.72) ranging from 0.03 to 3.24. The characteristics of the participants are shown in Table 1.

**Table 1:** Demographic and menstrual characteristics of the study sample (n = 602)

	With PMS (n = 602)	Without PMDD (n = 378)	With PMDD (n = 224)
	**No. (%)**	**No. (%)**	**No. (%)**

**Age groups (year)**			

14-15	265 (44)	172 (45.4)	93 (41.7)

16-17	311(52)	192(50.8)	119 (53)

18-19	26 (4)	14 (3.8)	12 (5.3)

Mean (SD)	15.76 (1.06)	15.70 (1.04)	15.86 (1.09)

**Age at menarche (year)**			

9-10	7 (1)	3 (0.8)	4 (1.8)

11-12	198 (33)	124 (32.7)	74 (32.9)

13-14	356 (59)	220 (58.2)	136 (60.8)

15-16	41 (7)	31 (8.3)	10 (4.5)

Mean (SD)	12.99 (1.13)	13.03 (1.16)	12.93 (1.07)

**Marital status**			

Single	541 (90)	340 (89.9)	201 (89.7)

Married	61 (10)	38 (10.1)	23 (10.3)

**Dysmenorrhea**			

Yes	505 (84)	307 (81.2)	198 (88.3)

No	97 (16)	71 (18.8)	26 (11.7)

**Severity of menstrual bleeding**			

Mild	38 (6.3)	25 (6.6)	13 (5.8)

Moderate	412 (68.4)	272 (71.9)	140 (62.3)

Severe	152 (25.3)	81 (21.5)	71 (31.8)

**Scores on the SCL-90-R**			

Mean (SD)	1.45 (0.72)	1.26 (0.57)	1.49 (0.82)

Range	0.03-3.24	0.34-2.27	0.03-3.24

All participants met the ICD-10 diagnostic criteria for PMS and 37.2% were suffering from PMDD according to the DSM-IV diagnostic criteria.

The comparisons of the SF-36 scores among students with and without PMDD are shown in Table 2. Students with PMDD scored significantly lower in all measures (P < 0.0001) except for physical functioning (P < 0.274) indicating that PMDD significantly affected their health-related quality of life. The differences were more evident on role emotional, role physical, social functioning and bodily pain.

**Table 2:** Comparison of the SF-36 scores in adolescents with and without PMDD*

	Without PMDD (n = 378)	With PMDD (n = 224)	
	**Mean (SD)**	**Mean (SD)**	**P**

**Physical functioning**	81.96 (20.20)	79.74 (21.87)	0.274

**Role physical**	74.35 (29.95)	52.27 (34.77)	< 0.001

**Bodily pain**	73.37 (20.04)	58.11(23.41)	< 0.001

**General health**	69.47 (15.88)	61.22 (16.02)	< 0.001

**Social functioning**	77.62 (19.84)	61.30 (21.24)	< 0.001

**Role emotional**	69.71 (35.12)	43.65 (38.29)	< 0.001

**Vitality**	64.33 (17.82)	53.92 (17.14)	< 0.001

**Mental health**	69.09 (16.85)	58.20 (18.70)	< 0.001

*Adjusted for age

## Discussion

The findings from this study showed that students with PMDD reported a poor health-related quality of life as measured by the SF-36. They specially reported poorer conditions on role emotional, role physical, social functioning and bodily pain. Similarly a recent publication using the SF-12v2 (the shortened version of the SF-36) found that women either at risk for PMS or PMDD were significantly more likely to report limitations than women with no indication of PMS in all health-related quality of life areas except for two physical functioning items and one mental health item and the general health item [15]. Although some investigators showed that that physical and mental premenstrual symptoms have similar significant impact on quality of life as measured by activities of daily life [16,17] or four of the five most prevalent premenstrual symptoms were found to be physical [18]; it is argued that physical symptoms are an important component of premenstrual syndromes, but these have been shown to be psychobiological in nature [19]. A study of the burden of premenstrual dysphoric disorder (PMDD) on health-related quality of life found that the burden of PMDD was greater on mental and emotional health-related quality of life domains than on physical health-related quality of life domains. The disease burden of PMDD on health-related quality of life was estimated by comparing SF-12v2 scores between women who were identified as being at risk for PMDD with those observed in the general U.S. female population [20].

Similar to the current study tow other investigations in Iran and Pakistan used the SF-36 to measure health-related quality of life in female adolescents who suffered from PMS and both found that quality of life was significantly lower in the affected group [21,22]. The former study compared quality of life in a sample of the second year high schoolgirls with and without PMS and found that there were only significant differences between those who suffered from severe PMS and healthy adolescents on mental health and vitality. The differences among healthy adolescents and those who suffered from mild and moderate PMS and for other measures on the SF-36 were not significant. It is argued that female adolescents with severe PMS might experience more stress and thus report poorer conditions. A study comparing women in two different groups (high and low PMS groups) revealed that women in the high PMS group had significantly more stress and poorer quality of life than women in the low PMS group [23]. However, in comparison with other studies participants in our study scored higher on all measures indicating that they had a better quality of life. These differences are difficult to interpret but might be due to different life styles, socioeconomic status or cultures [7,21-23].

The finding from this study also revealed that PMS was common among female students. All participants in the present study had reported at least one symptom of PMS. Similarly a study from Iran revealed that 98.2% of university students aged 18-27 years were experiencing at least one mild to severe premenstrual symptom [24]. Overall evidence suggests that PMS is a common disorder among Asian adolescents. A Turkish study reported that 61.4% of adolescent girls suffer from PMS [25] and Lee et al. also found that 76% of Chinese female undergraduates reported at least one premenstrual symptom [26]. A cross-sectional survey of 1,295 rural adolescent girls aged 13 to 19 years in Malaysia showed that most participants (63.1%) identified themselves as having premenstrual symptoms [27]. However agreement on using similar criteria and a standard measure to assess premenstrual disorders in adolescents might be a solution for the problem of such differences in reporting. As such a recent publication suggested that the Premenstrual Symptoms Screening Tool modified for adolescents (PSST-A) is a fast, and reliable tool to screen for these syndromes in adolescents [28]. For instance a study from Sri Lanak determining PMS by the PSST-A and American College of Obstetricians and Gynecologists (ACOG) diagnostic criteria reported that individual premenstrual symptoms were experienced by 65.7% of schoolgirls but the prevalence of PMS was only 8.75% [29].

A New developed instruments for evaluating the impact of premenstrual symptoms on HRQOL make the importance of our study more outstanding. The Premenstrual Symptoms Impact Survey (PMSIS), a web-based instrument administered to 971 women aged 18-45 years, revealed that the PMSIS scores differed between women with high, average or low health-related quality of life as measured by the SF-12 physical and mental component scores. Wallenstein and colleagues concluded that the PMSIS could be used to distinguish different clinical groups and assess the impact of symptoms on HRQOL [30].

### Limitations

There were some limitations inherent to this study. Firstly, all students reported at least one premenstrual symptom and thus according to the ICD-10 criteria we identified all as having premenstrual syndrome. One should be cautious that our data was self-reported and more importantly using different criteria might lead to different results. A study found that 54% of adolescents aged 13 to 18 years said that they had PMS but only 31% met the diagnostic criteria [31]. Secondly, using a generic measure to assess health-related quality of life among this population rather than using a disease-specific tool such as the Premenstrual Symptoms Impact Survey (PMSIS) makes the findings limited. In addition, school functioning is an important issue among adolescents. The SF-36 ignores such a dimension and one should use well-known instruments to assess health-related quality of life among children and adolescents (e.g. the Pediatric Quality of Life Inventory- PedsQL). However, at commence of the present study none of these instruments were available in Iran.

## Conclusion

The findings from this study confirmed that adolescents with PMS/PMDD suffer from a poor HRQOL. The negative effects on the perceived HRQOL in adolescents were more significant in those with PMDD. The findings suggest that health and educational authorities need to recognize the problem and provide appropriate, tangible and emotional support for female students with premenstrual disorders at schools especially for those who suffer from PMDD. In addition, there is need to establish and strengthen school-based reproductive health education programs to enable female students to learn how to deal with these disturbing problems. Indeed future research should focus primarily on implementation and evaluation of school-based health education programs on the topic.

## Competing interests

The authors declare that they have no competing interests.

## Authors' contributions

MD was the main investigator, collected the data and wrote the first draft. FG and PA were the study supervisors. SST, AK and AM were the study advisors. AM provided the final manuscript. All authors read and approved the final manuscript.
